# Betablockers and Ivabradine Titration According to Exercise Test in LV Only Fusion CRT Pacing

**DOI:** 10.3390/diagnostics12051096

**Published:** 2022-04-27

**Authors:** Cristina Vacarescu, Constantin-Tudor Luca, Horea Feier, Dan Gaiță, Simina Crișan, Alina-Gabriela Negru, Stela Iurciuc, Emilia-Violeta Goanță, Cristian Mornos, Mihai-Andrei Lazăr, Caius-Glad Streian, Diana-Aurora Arnăutu, Vladiana-Romina Turi, Dragos Cozma

**Affiliations:** 1 Cardiology Department, “Victor Babes” University of Medicine and Pharmacy, 2 Eftimie Murgu Sq., 300041 Timisoara, Romania; vacarescucristina@yahoo.com (C.V.); horea.feier@gmail.com (H.F.); dgaita@cardiologie.ro (D.G.); eivanica@yahoo.com (A.-G.N.); stela_iurciuc@yahoo.com (S.I.); ema.goanta@yahoo.com (E.-V.G.); mornoscristi@yahoo.com (C.M.); mihai88us@yahoo.com (M.-A.L.); cstr100@gmail.com (C.-G.S.); bordejevic.aurora@gmail.com (D.-A.A.); turi.vladiana@umft.ro (V.-R.T.); dragoscozma@gmail.com (D.C.); 2 Institute of Cardiovascular Diseases Timisoara, 13A Gheorghe Adam Street, 300310 Timisoara, Romania; 3 Research Center of the Institute of Cardiovascular Diseases Timisoara, 13A Gheorghe Adam Street, 300310 Timisoara, Romania

**Keywords:** fusion CRT pacing, exercise test, betablocker, ivabradine optimization

## Abstract

Background: Betablockers (BB)/ivabradine titration in fusion CRT pacing (CRTP) is understudied. Aim: To assess drug optimization using systematic exercise tests (ET) in fusion CRTP with preserved atrioventricular conduction (AVc). Methods: Changes in drug management were assessed during systematic follow-ups in CRTP patients without right ventricle lead. Shorter AVc (PR interval) allowed BB up-titration, while longer AVc needed BB down-titration, favoring ivabradine. Constant fusion pacing was the goal to improve outcomes. Results: 64 patients, 62.5 ± 9.5 y.o divided into three groups: shorter PR (<160 ms), normal (160–200 ms), longer (200–240 ms); follow-up 59 ± 26 months. Drugs were titrated in case of: capture loss due to AVc shortening (14%), AVc lengthening (5%), chronotropic incompetence (11%), maximum tracking rate issues (9%), brady/tachyarrhythmias (8%). Interventions: BB up-titration (78% shorter PR, 19% normal PR, 5% longer PR), BB down-titration (22% shorter PR, 14% normal PR), BB exclusion (16% longer PR), adding/up-titration ivabradine (22% shorter PR, 19% normal PR, 5% longer PR), ivabradine down-titration (22% shorter PR, 3% normal PR), ivabradine exclusion (11% normal PR, 5% longer PR). Drug strategy was changed in 165 follow-ups from 371 recorded (42% patients). Conclusions: BBs/ivabradine titration and routine ET during follow-ups in patients with fusion CRTP should be a standard approach to maximize resynchronization response. Fusion CRTP showed a positive outcome with important LV reverse remodeling and significant LVEF improvement in carefully selected patients.

## 1. Introduction

Left ventricle (LV) only pacing is a validated alternative to biventricular (BiV) pacing for patients with heart failure (HF) and CRT-P indication and may be considered to maximize resynchronization response [1]. Several randomized trials and studies demonstrated that LV-only pacing induces short-term hemodynamic benefits and a long and medium-term favorable response [2,3,4,5].

Left univentricular CRTP rationale strategy is to preserve intrinsic atrioventricular conduction in order to provide a fusion activation via the right bundle branch and the LV paced rhythm without the potential of additional desynchronization through RV pacing [6]. Exclusive fusion CRTP without RV lead showed a positive outcome in carefully selected patients [7,8]. Moreover, several studies showed that fusion pacing was associated with resynchronization super response, probably by shortening LV activation time [9,10].

LV-only pacing is mandatory in fusion CRTP. Of note, AV delay programming in all CRTP devices is influenced by AV conduction. However, AV conduction is physiologically related to physical exercise and heart rate, and can be highly influenced by medication such as betablockers, antiarrhythmics, and other heart rate modulation agents. Therefore, medication changes and programming in follow-ups is important, nevertheless, understudied in order to increase responsiveness to CRT.

The previous 2013 ESC cardiac pacing and resynchronization guideline stated that LV-only pacing CRT is non-inferior to BiV pacing. The current 2021 similar ESC guideline does not provide any directive regarding LV-only pacing, neither pro nor contra, which highlights an important gap of knowledge regarding this area [11,12].

Our assumption was that betablockers and ivabradine titration could play an important role in maintaining constant fusion pacing by careful optimization in order to obtain a convenient AV conduction. According to the PR interval, different drug management strategies have been approached: patients with a shorter PR interval permitted an increased dosage of BB (up-titrated to the maximum dose, when possible), while longer PR interval patients were provided with ivabradine without BB or a combination of lower dose BB.

The exercise test has proven to be an important tool for follow-up and optimization of CRT devices (13), specifically in fusion pacing [13,14]. The objective of this study was to assess the role and feasibility of BB/ivabradine titration using systematic exercise tests to obtain constant and permanent fusion pacing in patients with normal AV conduction. This article not only offers valuable data in a grey area of CRT, yet the results section was built as a step-by-step guideline in order to help peers identify the main issues associated with fusion pacing and to provide individualized solutions.

## 2. Materials and Methods

### 2.1. Study Population & Study Design

This study included patients in sinus rhythm with normal AV conduction implanted with CRT-P between 2011 and 2020 at the Timisoara Institute of Cardiovascular Diseases; the indication was in accordance with the available ESC 2013 guideline [11]. Fusion pacing CRT was of primary intention using a right atrium/left ventricle (RA/LV) DDD pacing system [7]. The inclusion criteria were: heart failure NYHA II-III, LBBB morphology with a QRS > 130 ms and preserved atrioventricular conduction (PR interval < 240 ms). Exclusion criteria for performing fusion RA/LV CRT-P were: AV block grade I with PR interval > 240 ms, AV block grade II/III, persistent atrial fibrillation or atrial fibrillation susceptibility documented by severe biatrial dilatation or a longer interatrial conduction time [15], confirmed coronary artery disease or history of myocardial infarction, syncope, CRT-D indication in secondary prevention, severe comorbidities (severe renal, hepatic failure, active neoplasia).

Systematic 6-month full follow-ups (ECG, clinical, echocardiography), including exercise tests, were recorded in all patients. A retrospective analysis was performed on evolution with a special focus on betablocker and ivabradine titration after each follow-up. Demographical, clinical, and paraclinical data were analyzed during 2011 (baseline data for the first patient with fusion pacing CRT) and June 2021 (last follow-up of this cohort group). ET data and consecutive drug management and CRT optimization were analyzed from 2014 to June 2021.

The study was conducted in accordance with the 1964 Helsinki Declaration and the protocol was approved by the Ethical Review Committee of the Timisoara Institute of Cardiovascular Diseases (1622/26 March 2014). Written informed consent was obtained from all patients included in the study.

### 2.2. Echocardiographic Evaluation

Complete transthoracic echocardiography evaluation was performed in all patients before CRT, the day after, and at 6-months follow-up intervals. A Vivid E9 echocardiograph (GE Health Medical, Milwaukee, WI, USA) and a 2.5 MHz transducer were used. Standard echocardiographic measurements were assessed according to echocardiography guidelines: left ventricular end-diastolic diameter (LVEDD), left ventricular end-diastolic volume (LVEDV), left ventricular end-systolic volume (LVESV), left ventricular ejection fraction (LVEF), left-atrial volume (LAV), assessment of valvulopathies and pulmonary systolic artery pressure (PSAP). Evaluation of dyssynchrony parameters involved assessment of atrioventricular, inter/intraventricular asynchronism, and septal flash. However, the Result section displays the most important data used to characterize the CRT response.

### 2.3. Exercise Test & Device Optimisation

A cycle-ergometry ET using a GE exercise system was performed systematically every 6-months. In the case of super response or patients with the previous ET without indication in reprogramming or drug optimization, the follow-up interval with ET was increased to 12 months. On the contrary, if fusion capture troubleshooting was detected, the ET was repeated immediately after reprogramming. Patients were rescheduled to be reassessed by exercise test no later than one month if drug optimization with significant dose changes was needed.

An adapted cyclo-ergometry Bruce protocol with an automatic 25 W workload increase at each 2 min exercise stage was used in all patients; exercise capacity was measured in metabolic equivalents of the task at peak exercise (METs). Blood pressure monitoring, permanent ECG evaluation, and live pacemaker interrogation were permanently recorded during the ET. CRT assessment evaluated maximal heart rate, beat-to-beat ECG analysis of true LV-fusion pacing, loss of LV capture, and improvement in exercise capacity with a special focus on maintaining constant fusion pacing during exercise. Any variations regarding the QRS morphology during exercise (with special focus on maintaining R wave in V1, V2) were followed by changes in dynamic AV interval in order to maintain a constant ECG aspect. In patients with chronotropic incompetence, the rate response function was programmed. In patients with capture loss due to exceeding the maximum tracking rate (MTR), a higher upper limit was programmed.

### 2.4. Betablocker and Ivabradine Titration Methodology

The patients were divided into 3 groups: the shorter PR group (<160 ms), normal PR group (160–200 ms), and longer PR group (200–240 ms). It is notable that any patients from the longer PR group with unstable AV conduction over 240 ms were excluded from the study. The feasibility of including patients with PR between 200–240 ms was based intuitively on our practical observation during decades of classical CRT follow-up [16]. Shorter PR interval patients permitted increased dosage of BB alone, while longer PR interval patients were provided with ivabradine without BB or a combination of lower dose BB. Overall medication was adapted individually to each patient and included either fixed combinations of BB/ivabradine or various doses of BB and/or ivabradine. The difference between the start of treatment and long-term follow-up medication was analyzed.

### 2.5. Statistical Analysis

Data were expressed as means ± SD for continuous variables and proportions for categorical variables. Continuous variables were compared between groups using unpaired t-tests (variables with normal distribution) or Mann–Whitney U tests (abnormally distributed variables). Proportions were compared using χ2 and Fisher’s exact test. *p* < 0.05 was considered significant. All analyses were carried out with the SPSS version 18.0 (SPSS Inc., Chicago, IL, USA).

## 3. Results

### 3.1. Baseline Clinical Data, Echocardiography, Device Programming, and Medication

The study included 64 patients, aged 62.5 ± 9.5 years, 35 males (55%), 29 females (45%), NYHA class II-III with LBBB morphology and QRS > 130 ms. At baseline, the mean EF was 26.6 ± 5.1%. The main demographic, echocardiographic, and ECG characteristics are presented in Table 1.

No acute or long-term complications (including lead/pocket infections) were noted regarding the pacemaker implant. LV lead position was postero-lateral in 38% of patients, lateral in 32% of patients, posterior in 11% of patients, anterolateral in 14% of patients, and epicardial in 5% of patients. At baseline, all the devices were programmed DDD 60 b/min with a mean AV pace of 148.5 ± 20 ms and a sensed AV delay of 119.9 ± 24 ms that allowed fusion pacing in all patients. The maximum tracking rate was set at the nominal value of 130 b/min for all the patients.

Three months of HF optimal medical therapy was mandatory before CRT and was continued individualized according to clinical and paraclinical variables (heart rate, blood pressure, renal function). Baseline medication, highlighting betablockers and ivabradine, are exposed in Table 2. Patients in the short and normal PR group received, at baseline, BBs and ivabradine in variable combinations, depending on the resting heart rate (HR) documented in the hospital (target 50–70 b/min). Patients in the longer PR group received mainly ivabradine or a small dose of BB, when needed, depending on the resting HR.

For a better understanding of our approach regarding the role of exercise tests in optimizing drug therapy, a step-by-step guide with different strategies is presented in Section 3.2.

### 3.2. Exercise Test Issues and Drug Management Solutions

#### 3.2.1. Strategy for Capture Loss Due to AV Conduction Shortening

**Figure 1:**
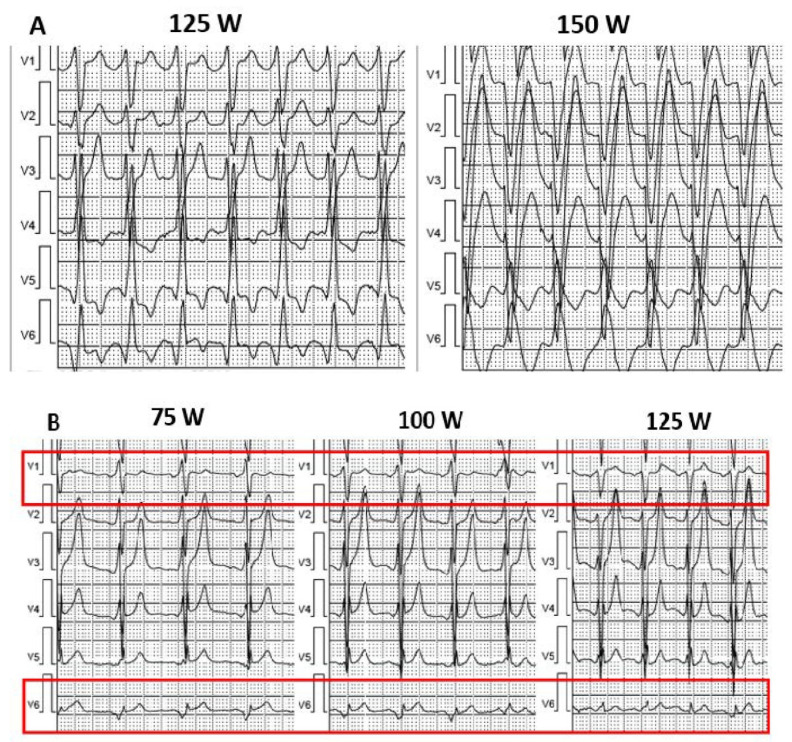
(**A**) Fusion pacing at 125 W: note the thin QRS complexes with r wave in V1 and q wave in V6. Complete loss of LV capture at 150 W: larger QRS with typical LBBB morphology. (**B**) Incomplete loss of capture: during exercise, a progressive loss of R wave in V1, V2, and q wave in V6 can be noted.

- Problem: physiological AV conduction shortening during exercise leads to complete or incomplete loss of ventricular capture (Figure 1A,B).
- Incidence: higher in patients with shorter PR interval (5 pts with shorter PR interval vs. 4 pts with normal PR vs. 0 pts with longer PR); the overall incidence in 9 patients (14%).
- Solution: patients with shorter PR were up-titrated to the maximum betablocker dose; patients with normal PR received a higher dose of betablocker + ivabradine.

#### 3.2.2. Strategy for Complete LV Capture without Fusion Due to AV Conduction Lengthening

**Figure 2:**
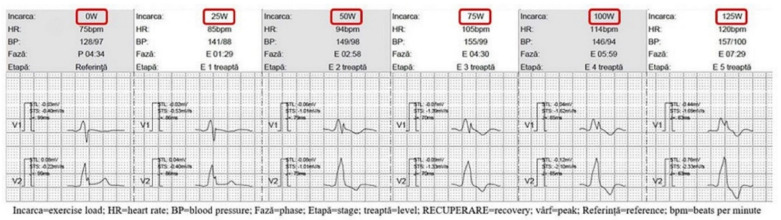
During exercise, a lengthening of PR interval leads to complete LV capture—RBBB appearance in V1, V2.

- Problem: during exercise, a lengthening of the PR interval was noted; consequently, this leads to a RBBB morphology due to maximum preexcitation of the LV (Figure 2).
- Incidence: higher in patients with longer PR interval (2 patients with longer PR interval vs. 1 patient with normal PR interval vs. 0 pt with shorter PR interval); overall incidence 3 patients (5%).
- Solution: BBs were excluded in patients with a longer PR interval and down-titrated in patients with a normal PR interval. The ivabradine dose was adjusted according to the maximum heart rate achieved during the exercise test.

#### 3.2.3. Strategy for Chronotropic Incompetence

**Figure 3:**
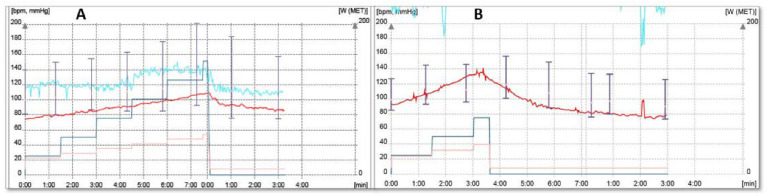
(**A**) Heart rate diagram (red line) during exercise shows a small and slow increase in heart rate during prolonged exercise. (**B**) One month after medication optimization, the ET was repeated, showing a good HR acceleration during exercise.

- Problem: several cases of chronotropic incompetence (Figure 3A,B) were exposed by exercise tests. Maximum heart rate (HR) and heart rate diagrams were the main diagnosis parameters. Although it may seem a trivial finding, chronotropic incompetence leads to a reduced cardiac outflow and low exercise tolerance.
- Incidence: higher in patients with normal PR (4 patients with normal PR vs. 2 patients with shorter PR vs. 1 patient with longer PR); overall incidence 7 patients (11%).
- Solution: BB and ivabradine doses were down-titrated in patients with shorter PR; ivabradine was excluded in patients with normal PR ± down-titration of BBs after the redo ET; for the patient with longer PR ivabradine was excluded.

#### 3.2.4. Strategy for Maximum Tracking Rate Issues

- Problem: a HR above the MTR during exercise leads to a complete loss of LV capture.
- Incidence: higher in patients with normal PR (2 patients with shorter PR vs. 3 patients with normal PR vs. 1 patient with longer PR); overall incidence 6 patients (9%).
- Solution: patients with shorter PR and normal PR were up-titrated to the maximum BB dose + maximum dose of ivabradine; patients with longer PR were up-titratred to the maximum dose of ivabradine and a small dose of BB.

#### 3.2.5. Strategy for Brady/Tachyarrhythmias during ET

**Figure 4:**
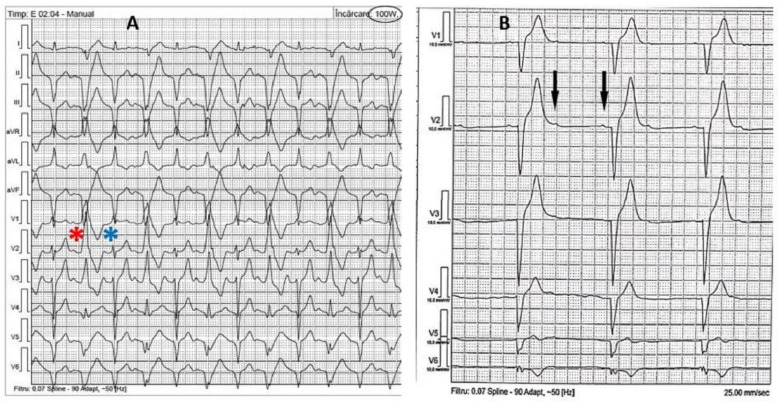
(**A**) ECG monitoring during ET: at 100 W, an alternative QRS morphology with narrow QRS (fusion pacing—blue asterisk) and large QRS (RBBB morphology due to complete LV capture—red asterisk) can be seen. (**B**) At pacemaker inhibition, a 2:1 AV block can be noted.

- Problem: episodes of paroxysmal atrial fibrillation (AF) were noted during maximum exercise in several patients. Also, a particular ECG aspect during ET revealed conduction disorders such as 2:1 AV block (Figure 4A,B).
- Incidence: incidence of paroxysmal atrial fibrillation 4 patients (6%); conduction disorders incidence 1 patient (2%). This patient, in the longer PR group, received previous amiodarone and BB treatment for paroxysmal AF episodes at device interrogation.
- Solution: amiodarone was initiated for patients with AF; all bradycardic therapy was excluded in the patient with AV block and upgrade to a triple chamber device was performed.

### 3.3. Follow-Up Data and Drug Management Analysis

The average follow-up was 59 ± 26 months. Thirty-one patients (48%) were implanted before 2016 and had more than 5 years of follow-up. Comparative data regarding the main clinical and echocardiographic parameters at baseline versus follow-up are presented in Table 3. Non-sudden cardiac death occurred in 6 patients (9%), both patients with constant and permanent fusion pacing (3%) and patients who needed device and medication optimization (6%): 3 patients developed refractory HF, while the other 3 died due to pulmonary sepsis (complicated pneumonia with late hospital presentation).

During total follow-up, atrial fibrillation incidence was 16%: 5 patients (8%) developed paroxysmal AF during ET or noted at device interrogation, 3 patients with persistent AF (5%), 2 patients (3%) with permanent AF. Amiodarone was initiated in patients with persistent and paroxysmal AF. Pulmonary vein isolation was performed in one patient with paroxysmal AF, and cavotricuspid isthmus ablation was performed for a patient (2%) with typical atrial flutter. No episodes of ventricular tachycardia were noted during follow-up and CRT-D upgrade was not necessary in any of our patients. A high number of premature ventricular beats resulted in a low percentage of pacing (<95%) in 2 patients: for one of them (RVOT origin) ablation was performed successfully, for another patient (LVOT origin) ablation was not possible due to an associated Leriche syndrome and amiodarone was initiated with a moderate increase in the pacing percentage (from 91 to 95%).

**Figure 5:**
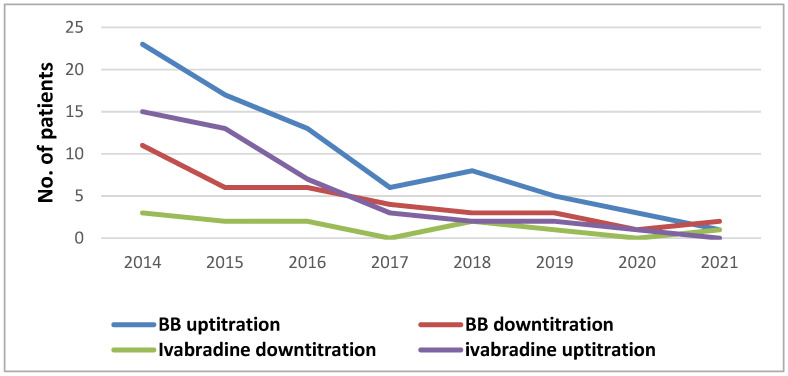
Graphical representation of drug management—number of interventions in medication/year.

A total of 371 ETs were done between 2014 and 2021. An adapted cyclo ergometry Bruce protocol was used in all patients, with a mean of 109 ± 35 W (6.1 ± 1.4 metabolic equivalents of task) and a peak heart rate of 115 ± 19 beats/min. The following drug interventions were needed: BB up-titration (78% shorter PR pts, 19% normal PR pts, 5% longer PR pts), BB down-titration (22% shorter PR pts, 14% normal PR pts), BB exclusion (16% longer PR pts), adding/up-titration ivabradine (22% shorter PR pts, 19% normal PR pts, 5% longer PR pts), ivabradine down-titration (22% shorter PR pts, 3% pts with normal PR), ivabradine exclusion (11% normal PR pts, 5% longer PR pts). In total, medication changes were performed in 165 follow-ups (44%). Detailed changes and a drug management analysis are presented in Figure 5 and Table 4. The graphical representation in Figure 5 reflects the number of interventions in medication/year.

An individualised device optimisation was also performed to ensure constant fusion pacing. The rate response function was programmed in patients with chronotropic incompetence (11%) and the MTR was increased to 145 b/min in 9% of patients. Dynamic AV interval was adapted in patients with AV conduction lengthening or shortening (overall incidence, 19%). A paced AV interval of 150.6 ± 31.2 ms (versus 148.5 ± 20 ms at baseline) and a sensed AV interval of 106.8 ± 25.7 ms (versus 119.9 ± 24 ms at baseline) allowed optimal fusion pacing in all patients.

At the end of follow-up, medication optimization was performed in 42% of patients, while CRT device reprogramming was needed in 39% of patients. The main outcome of this systematic follow-up was a 90% responder rate in our cohort associated with an important reverse remodelling process regarding LV end-systolic volume (190.1 ± 81.7 vs. 137.4 ± 82.1, *p* = 0.0006), LV end-diastolic volume (244.7 ± 86.4 vs. 210.5 ± 84, *p* = 0.0028) and LV ejection fraction (26.6 ± 5.1 vs. 37.2 ± 9.5, *p* < 0.0001).

## 4. Discussion

This study presents new real-life information regarding fusion CRT-P drug optimization during systematic follow-ups using ET as a tool to detect troubleshooting in efficient pacing. The outcome of the medication strategy used in our study is positive for a population with preserved AV conduction. To the best of our knowledge, this is the first study to describe in detail a complete strategy to increase the responsiveness of CRT. In addition, the results confirm the need for careful and systematic follow-ups with ETs, drug, and device optimization.

Variability of AV conduction is one of the main concerns regarding the target of more than 95% pacing in CRT. To ensure the stability of AV conduction in our population, we used drug optimization and CRT reprogramming during follow-ups. Thus, the result was better maintenance of a “stable” QRS duration and morphology, both at rest and during exercise. A significant reduction in QRS duration was achieved (160.6 ± 16 ms at baseline vs. 130.9 ± 9.4 at follow-up, *p* < 0.0001). Several studies showed that the magnitude of QRS duration narrowing after CRT was associated with improved clinical response, while fusion pacing CRT has typically shown shorter paced QRS duration, making sense with our study strategy and results [17,18,19,20,21,22].

With long-lasting evidence, betablockers and ivabradine are part of the current standard treatment in chronic HF [23]; however, inconsistent dose titration, especially regarding BBs and ACE-i, is common in clinical practice [24]. Several studies evaluated BB optimization post resynchronization and found a positive correlation between a favorable CRT outcome and dose up-titration [25,26]. The main reason to up-titrate BBs in this study was to achieve the intrinsic AV conduction stability. Nevertheless, after a mean follow-up of 59 ± 26 months, there was an increase in the number of patients treated and the dosage administered in the majority of patients. The variability of AV conduction was documented in both ways: shorter PR interval during physical exercise due to increased heart rate and the subsequent need to program rate-adaptive AV interval and increased intrinsic PR interval after resynchronization, irrespective of the CRT response status [27,28]. In the current study group, the main reasons for avoiding high BB doses were a longer baseline PR interval or a documented prolonged AV conduction during ET.

Another observation during follow-up was the association between high doses of BB and chronotropic insufficiency with reduced physical exercise tolerance. Our primary choice was ivabradine, with or without a small dose of beta-blocker, in these categories of patients, depending on the baseline PR and the AV conduction behavior. Although the number of patients treated with ivabradine is similar at baseline compared with the follow-up data (52% vs. 48%), the dose used reached a statistical significance (11.1 ± 2.4 mg at baseline vs. 12.3 ± 1.2 mg during follow-up, *p* = 0.0195), suggesting that a higher dose of ivabradine could represent the treatment of choice for a selected category of patients. In patients with shorter PR, BB up-titration was the main choice, providing both stability of the AV conduction and optimal heart rate control. Adding ivabradine was the second option in short PR patients with inadequate heart rate control during maximum exercise.

The drug management with three different strategies according to the PR interval (shorter, normal, and longer PR) was based on direct observation in the early years of fusion pacing CRT in our clinic and was systematically approached in all patients between 2014 and 2021. BBs were the most titrated depending on the PR variability during exercise, especially in the normal and shorter PR group. An interesting finding was noted in the longer PR group, where a lower rate of optimizations after ET was needed to achieve stability of fusion pacing (5 pts in the longer PR group vs. 22 pts in the normal and shorter PR group), encouraging fusion pacing CRT in patients with a slightly longer AV conduction (PR 200–240 ms). Another valuable piece of information can be extracted from the graphical representation of drug management during follow-up (Figure 5), proving that a higher number of adjustments was needed in the first years after performing fusion CRT-P, with a significant decrease in mid and long term follow-up. These findings suggest that if individualized optimizations are fulfilled, and a stable clinical condition is reached, constant fusion pacing can be provided without further medication or device programming changes.

A possible limitation of this study is the relatively small number of patients included and the unequal distribution between the three subgroups. Another potential limitation is the high number of responders in our cohort (90%); however, this is due to a strict selection (non-ischemic cardiomyopathy, typical LBBB, QRS > 130 ms) and to a careful and tailored follow-up for each patient. Every six months, follow-ups including an exercise test, echocardiography, personalized device reprogramming, and medication optimization provided the “best to get” therapy for each individual patient, maximizing the overall CRT response. Nevertheless, according to a recent review prepared on behalf of the EHRA Education Committee [1], LV only pacing by itself may lead to a better CRT outcome and may decrease the number of non-responders.

A recent review addresses a similar approach regarding fusion pacing as a method for improving patients outcome in CRT; a future clinical perspective should take into account that promoting fusion pacing with integrated novel techniques such as multipoint pacing or dedicated algorithms could increase the number of CRT responders [29]. The main criticism of using a dual-chamber device in order to provide cardiac resynchronization would be the absence of the defibrillation function. According to the past 2013 and the current 2021 ESC pacing and cardiac resynchronization therapy guidelines [11,12], CRT-D therapy is required in secondary prevention and would be more appropriate in younger patients with ischemic heart disease; these patients were excluded from our study population. Large randomized trials have brought consistent data regarding this subject. The Danish trial showed that prophylactic ICD implantation in patients with symptomatic systolic heart failure not caused by coronary artery disease was not associated with a significantly lower long-term rate of death from any cause than was usual clinical care [30]. Moreover, the recent RESET-CRT project, which analyzed a large database of CRT implantations, demonstrated no survival differences between CRT-D and CRT-P after adjustment for age and entropy balancing [31].

We emphasized that this observational retrospective study can lay the foundation for a randomized trial to assess fusion pacing CRT in multiple PR interval subcategories and different drug management strategies to stabilize AV conduction. This paper brings valuable data in the scarce area of fusion pacing, thus helping future guidelines and industry by cost-efficiently providing better and individualized CRT options.

## 5. Conclusions

Betablockers and ivabradine titration during follow-ups in patients with fusion CRT pacing should be a standard approach additional to routine exercise tests in order to maximize resynchronization response. Fusion CRT-P showed a positive outcome with important LV reverse remodelling and significant LVEF improvement in carefully selected patients.

## Figures and Tables

**Table 1:** Demographic, ECG, and echocardiographic baseline data—at the moment of pacemaker implantation.

	All Patients (N = 64)	
**Demographic baseline data**	Male gender, N, %	35 (55%)
	Female gender, N, %	29 (45%)
	Age, y.o, mean ± SD	62.5 ± 9.5
	HF NYHA II, N, %	32 (50%)
	HF NYHA III, N, %	32 (50%)
	**Associated pathology, N, %**	
	Hypertension	26 (41%)
	CKD	30 (47%)
	Diabetes Mellitus	26 (41%)
	COPD	16 (25%)
**ECG characteristics**		
	QRS duration, ms, mean ± SD	160.6 ± 16
	PR interval, ms, mean ± SD	186.7 ± 32.4
	**PR interval groups, N, %**	
	Shorter PR (<160 ms)	9 (14%)
	Normal PR (160–200 ms)	36 (56%)
	Longer PR (200–240 ms)	19 (30%)
**Echocardiographic baseline data**		
	LVEF, %, mean ± SD	26.6 ± 5.1
	**Mitral regurgitation,** **N, %**	
	Mild	5 (8%)
	Moderate	26 (41%)
	Severe	33 (51%)
	LVEDV, mL, mean ± SD	244.7 ± 86.4
	LVESV, mL, mean ± SD	190.1 ± 81.7
	LAV, mL, mean ± SD	101.6 ± 32.8
	PSAP, mmHG, mean ± SD	45.8 ± 15.2

CKD = chronic kidney disease, COPD = chronic obstructive pulmonary disease, LVEF = ejection fraction, HF = heart failure, LAV = left atrium volume, LVEDV = left ventricle end diastolic volume, LVESV = left ventricle end systolic volume, N = number of patients, PSAP = pulmonary systolic artery pressure, SD = standard deviation.

**Table 2:** Baseline medication—at the moment of pacemaker implantation.

Class of Medication	Drug Name	Mean Dose ± SD (mg)	N and % of Patients
Betablocker	Metoprolol succinate	78.5 ± 28.4	22 (34%)
	Carvedilol	20.1 ± 12.2	19 (30%)
	Nebivolol	5	2 (3%)
	Bisoprolol	6.2 ± 3.2	8 (13%)
I*_f_* channel inhibitor Diuretics ACE-i Ang-II receptor blockers ARNI (fixed combination of sacubitril/valsartan)	Ivabradine Furosemid Spironolactone Zofenopril Ramipril Perindopril Candesartan Irbesartan	11.1 ± 2.4 72.8 ± 54.1 42.8 ± 9.5 14.2 ± 8.7 3.9 ± 3.2 5.6 ± 3.1 9.14 ± 6 225 ± 53.4 32 ± 13.8/34.6 ± 15	33 (52%) 64 (100%) 57 (89%) 20 (31%) 6 (9%) 5 (8%) 21 (33%) 3 (5%) 5 (8%)
SGTL2 inhibitors	Dapaglifozin	10	11 (17%)

ACE-i = angiotensin-converting enzyme inhibitors, Ang-II = angiotensin II, ARNI = angiotensin receptor–neprilysin inhibitor, I*_f_* = I funny, N = number, RAAS = renin–angiotensin–aldosterone system, SD = standard deviation.

**Table 3:** Comparative data regarding the main clinical and echocardiographical parameters at baseline versus follow-up.

	Baseline Data N = 64 pts	Follow-Up Data (59 ± 26 Months) N = 58 pts	% of Relative Change *	*p* Value
Pts in NYHA class III, N, %	32 (50%)	5 (9%)	−84%	-
Pts in NYHA class II, N, %	32 (50%)	31 (53%)	+3%	-
Pts in NYHA class I, N, %	-	22 (38%)	-	-
LVEF, mean ± SD	26.6 ± 5.1	37.2 ± 9.5	+28%	**<0.0001**
LVEDV (mL), mean ± SD	244.7 ± 86.4	210.5 ± 84	−14%	**0.0288**
LVESV (mL), mean ± SD	190.1 ± 81.7	137.4 ± 82.1	−28%	**0.0006**
Severe mitral regurgitation, N, %	33 (51%)	16 (28%)	−52%	-
QRS duration, ms, mean ± SD	160.6 ± 16	130.9 ± 9.4	−18%	**<0.0001**

* value of relative change calculated as a percentage.

**Table 4:** Drug management analysis: baseline data versus follow-up data.

	Baseline Data N = 64 pts		Follow-Up Data N = 58 pts		*p* Value *	% of Relative Change **
	Mean Dose ± SD (mg)	N and % of Patients	Mean Dose ± SD (mg)	N and % of Patients		
Metoprolol succinate	78.5 ± 28.4	22 (34%)	112.2 ± 36.7	28 (48%)	**0.0009**	+21%
Carvedilol	20.1 ± 12.2	19 (30%)	21.8 ± 15.9	11 (19%)	0.7446	−42%
Nebivolol	5	2 (3%)	5	4 (7%)	-	+50%
Bisoprolol	6.2 ± 3.2	8 (13%)	6.1 ± 2.9	10 (17%)	0.9455	+20%
Ivabradine	11.1 ± 2.4	33 (52%)	12.3 ± 1.2	28 (48%)	**0.0195**	−15%

* *p* value regarding the dosage; ** % of relative change regarding the number of patients treated.
